# CD1d-Dependent iNKT Cells Control DSS-Induced Colitis in a Mouse Model of IFNγ-Mediated Hyperinflammation by Increasing IL22-Secreting ILC3 Cells

**DOI:** 10.3390/ijms22031250

**Published:** 2021-01-27

**Authors:** Hyun Jung Park, Sung Won Lee, Luc Van Kaer, Seokmann Hong

**Affiliations:** 1 Department of Integrative Bioscience and Biotechnology, Institute of Anticancer Medicine Development, Sejong University, Seoul 05006, Korea; 0402parkhj@gmail.com (H.J.P.); insiderjjang@gmail.com (S.W.L.); 2 Department of Pathology, Microbiology and Immunology, Vanderbilt University School of Medicine, Nashville, TN 37232, USA; luc.van.kaer@vumc.org

**Keywords:** iNKT cells, CD1d, IFNγ, ILC3, IL22

## Abstract

We have previously shown that CD1d-restricted iNKT cells suppress dysregulated IFNγ expression and intestinal inflammation in Yeti mice on the C57BL/6 background. Since type 3 innate lymphoid cells (ILC3s) in mesenteric lymph nodes (MLN) protect against intestinal inflammation in a CD1d-associated manner, we investigated whether crosstalk between iNKT cells and MLN ILC3s controls IFNγ-mediated intestinal inflammation in Yeti mice. We found that Yeti mice display increased levels of ILC3s and that iNKT cell deficiency in Yeti/CD1d KO mice decreases levels of IL22-producing ILC3s during DSS-induced colitis. This finding indicates that iNKT cells and ILC3s cooperate to regulate intestinal inflammation in Yeti mice. Yeti iNKT cells displayed a pronounced anti-inflammatory (IL4- or IL9-producing) phenotype during colitis. Their adoptive transfer to iNKT cell-deficient animals induced a significant increase in IL22 production by ILC3s, indicating that crosstalk between iNKT cells and ILC3s plays a critical role in modulating colitis in Yeti mice. Moreover, we showed that the IL9-producing subset of iNKT cells potently enhances IL22-producing ILC3s in vivo. Taken together, our results identify a central role of the iNKT cell-ILC3 axis in ameliorating IFNγ-mediated intestinal inflammation.

## 1. Introduction

Inflammatory bowel disease (IBD) is a chronic inflammatory disorder that causes inflammation in the digestive system. The two major forms of IBD are Crohn’s disease and ulcerative colitis, which are characterized by intestinal epithelial barrier breakdown [1]. The experimental IBD model induced with dextran sulfate sodium (DSS) closely mimics many aspects of Crohn’s disease, including epithelial damage, edema, and crypt damage [2]. The pro-inflammatory cytokine interferon-gamma (IFNγ) plays a critical role in DSS-induced colitis. For example, mice with an IFNγ gene ablation are protected against DSS-induced colitis [3]. In contrast, Yeti mice with prolonged expression of IFNγ via modification of their 3′ untranslated region (UTR) exhibit increased susceptibility to DSS-induced colitis [4]. In addition to IFNγ, commensal bacteria contribute to this inflammatory process due to breakdown of the intestinal epithelial barrier elicited by DSS treatment. Stimulation of mononuclear phagocytes by commensal bacteria leads to production of both pro- and anti-inflammatory cytokines, indicating the presence of a homeostatic mechanism to regulate inflammatory immune responses [5].

Natural killer T (NKT) cells are a heterogeneous group of T cells that express αβ T cell receptors and various NK cell surface markers such as NKR-P1 (NK1.1). Invariant NKT (iNKT) cells express a semi-invariant T cell receptor (Vα14Jα18 in mice and Vα24Jα18 in humans) specific for glycolipid antigens bound with the major histocompatibility complex (MHC) class I-related protein CD1d [6,7]. Because iNKT cells can produce various cytokines, they play either protective or pathogenic roles in immune-related diseases [8,9,10], including the development of colitis. For example, adoptive transfer of iNKT cells exerted protective effects against DSS-induced colitis [11]. Our previous study supports these protective aspects of iNKT cells in intestinal immune responses by demonstrating that iNKT cell deficiency in mice increases susceptibility to IFNγ-mediated intestinal inflammation [4].

Type 3 innate lymphoid cells (ILC3s), an ILC subset expressing the transcription factor RORγt, participate in downregulating colonic inflammation by inhibiting commensal bacteria-reactive T cells, in a mechanism involving ILC3 expression of MHC class II [12]. Moreover, ILC3s are the primary producer of the cytokine IL22, which is necessary for protecting and recovering the intestine from colitis [13]. They are also the main ILC subset within mucosal draining lymph nodes (LN), such as mesenteric LNs (MLNs) [14]. Interestingly, Saez de Guinoa et al. reported that activation of IL22-producing ILC3s is mediated by CD1d, suggesting potential crosstalk between ILC3s and CD1d-dependent iNKT cells [15].

We have previously demonstrated that CD1d-dependent iNKT cells play a significant role in controlling DSS-induced colitis in C57BL/6 (B6) background Yeti mice with dysregulated IFNγ-mediated autoinflammation [4]. However, it is unclear whether iNKT cells and ILC3s cooperate to control mucosal immune responses during DSS-induced colitis in these animals. To address this issue, we performed adoptive transfer experiments to compare the effects of MLN iNKT cells purified from either wild-type (WT) or Yeti mice on ILC3 activation in recipient iNKT cell-deficient mice during DSS colitis. We found that adoptive transfer of Yeti iNKT cells induced a significant increase of IL22 production by ILC3s in the spleen and MLN of recipient animals. Furthermore, we found that IL9-producing iNKT cells were closely linked with the expansion of IL22-producing ILC3s. From these findings, we conclude that iNKT cells and ILC3s cooperate to suppress colitis in Yeti mice.

## 2. Results

### 2.1. Yeti Mice Display a Dramatic Increase of IL22-Producing ILC3s during DSS-Induced Intestinal Inflammation

Yeti mice on the C57BL/6 (B6) genetic background (hereafter referred to as Yeti mice) display autoinflammatory syndromes due to abnormal IFNγ secretion [3,4,16,17]. Consequently, these animals spontaneously develop colitis when housed under conventional, but not barrier, conditions, and develop exacerbated colitis in response to DSS treatment. We recently reported that NK1.1^+^CD8^+^ (CD1d-independent) T cells are critical effector cells that exacerbate DSS-induced colitis in Yeti mice [4,18]. Using adoptive transfer studies in RAG2 KO recipient animals, we confirmed that CD8^+^ T cells rather than CD4^+^ T cells are the relevant colitis effector cells in Yeti mice (Figure S1). However, we also detected a significant increase in anti-inflammatory cytokines such as IL4, IL9, IL22, and TGFβ in Yeti MLN cells compared with WT MLN cells after DSS treatment, suggesting a regulatory pathway that compensates for detrimental pro-inflammatory cytokine production in the intestine of Yeti mice (Figure S2).

**Figure 1:**
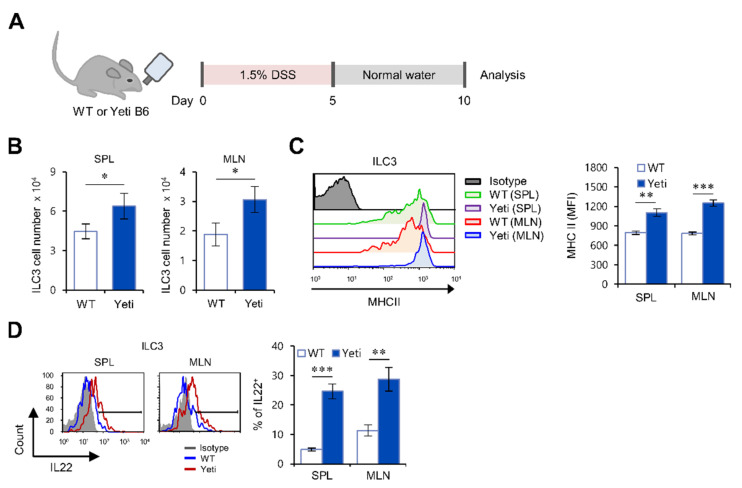
Dysregulated expression of interferon-gamma (IFNγ) increases IL22-producing type 3 innate lymphoid cells (ILC3) in dextran sulfate sodium (DSS)-treated Yeti mice. (**A**) Experimental outline. Wild-type (WT) and Yeti mice were treated with 1.5% DSS as described in Materials and Methods. The spleen and mesenteric lymph nodes (MLN) were collected ten days after initiation of DSS treatment. (**B**) The absolute numbers of ILC3s (MHC II^+^RORγt^+^Lin^−^ (i.e., CD3, CD19, NK1.1, CD11b, B220, F4/80, CD11c, Gr1, FcεR1α)) in the spleen and MLN from WT and Yeti mice were assessed by flow cytometry. (**C**) MHC II expression by splenic and MLN ILC3s (RORγt^+^Lin^−^) from these mice was analyzed by flow cytometry. Representative data (left panel) and their summary (right panel) are shown. (**D**) IL22 production by splenic and MLN ILC3s (MHC II^+^RORγt^+^Lin^−^) from these mice was determined by flow cytometry on day 10. The mean values ± SD (*n* = 4; per group in the experiment; Student’s *t*-test; * *p* < 0.05, ** *p* < 0.01, *** *p* < 0.001) are shown. One representative experiment of two experiments is shown.

The cytokine IL22 participates in wound healing during intestinal inflammation. Since ILC3s are the predominant cellular source of IL22 in the MLN [19], we examined whether ILC3s play a role in maintaining intestinal homeostasis in the context of IFNγ-mediated inflammation in Yeti mice. To explore this possibility, we compared the frequency, MHC II expression, and IL22 production of ILC3s in the MLN between Yeti and WT mice after DSS treatment. We found that Yeti mice exhibited a significant increase in the absolute cell number and MHC II expression of ILC3s from both the spleen and MLN during DSS-induced colitis (Figure 1A–C). Furthermore, we found that IL22 production by ILC3s in the spleen and MLN of Yeti mice was approximately fourfold higher than WT mice (Figure 1D). Taken together, these findings suggest that IL22-producing ILC3s suppress DSS-induced acute colitis in Yeti mice.

### 2.2. The Frequency and Function of ILC3s in the MLN of Yeti Mice Are Dependent on CD1d-Dependent iNKT Cells

**Figure 2:**
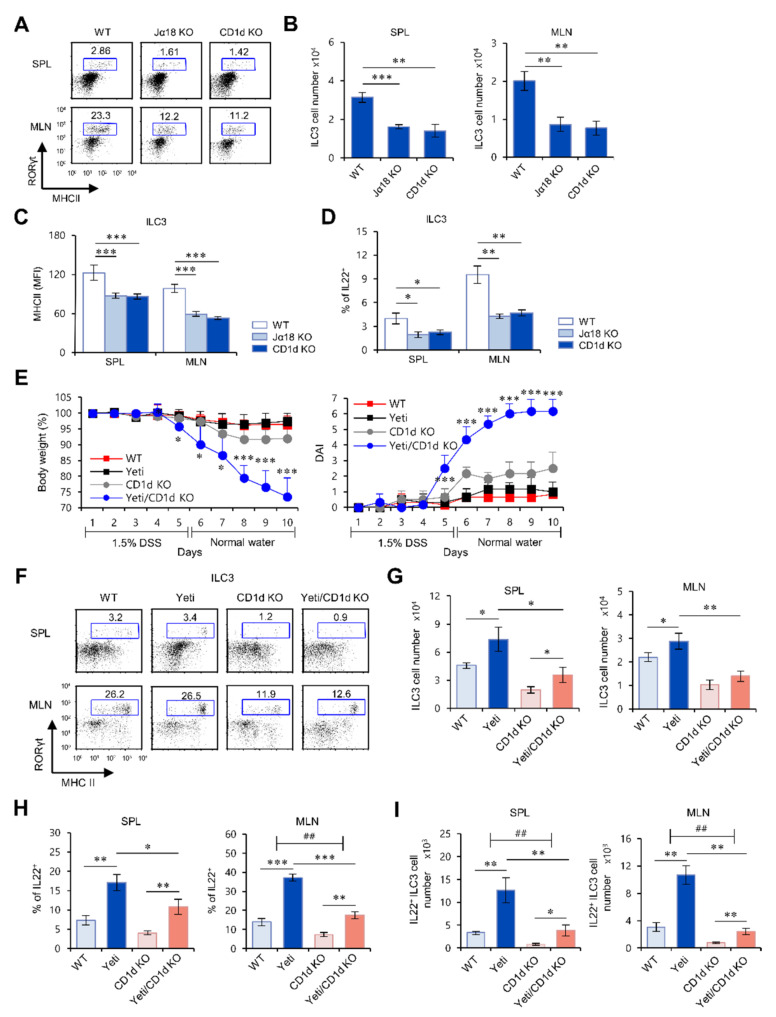
The frequency and function of ILC3s in the MLN of Yeti mice are dependent on CD1d-dependent iNKT cells. (**A**–**D**) The spleen and MLN were prepared from WT, Jα18 KO, and CD1d KO mice at six weeks of age. (**A**,**B**) The absolute numbers of ILC3s (RORγt^+^MHC II^+^Lin^−^) in the spleen and MLN from these mice were assessed by flow cytometry. Representative data (**A**) and their summary (**B**) are shown. (**C**) MHC II expression by splenic and MLN ILC3s (RORγt^+^Lin^−^) from these mice was determined by flow cytometry. (**D**) IL22 production by splenic and MLN ILC3s (MHC II^+^RORγt^+^Lin^−^) from these mice was analyzed by flow cytometry. (**E**–**I**) Daily body weight changes and DAI scores of WT, Yeti, CD1d KO, and Yeti/CD1d KO mice were evaluated after 1.5% DSS treatment. (**F**,**G**) The absolute numbers of ILC3s (MHC II^+^RORγt^+^Lin^−^) in the spleen and MLN from these mice were assessed by flow cytometry ten days after DSS treatment. (**F**) Representative FACS plots and (**G**) summary. (**H**) IL22 production by splenic and MLN ILC3s (MHC II^+^RORγt^+^Lin^−^) from these mice was determined by flow cytometry on day 10. (**I**) The number of IL22-producing ILC3s among these cells was assessed by flow cytometry on day 10. The mean values ± SD (*n* = 5; per group in the experiment; Student’s *t*-test; * *p* < 0.05, ** *p* < 0.01, *** *p* < 0.001) are shown. Two-way ANOVA (Yeti × iNKT) showed an interaction between these two factors (^##^ *p* < 0.01). One representative experiment of two experiments is shown.

iNKT cells can be activated by glycolipid ligand, α-GalCer, loaded on CD1d expressed on ILC3s [15]. Thus, to test whether ILC3s can be regulated by iNKT cells, we next examined whether ILC3s are altered in CD1d KO and Jα18 KO mice, which both lack iNKT cells. Interestingly, we found a significant decrease in cell number, MHC II expression, and IL22 production of ILC3s in both CD1d KO and Jα18 KO mice compared with WT mice (Figure 2A–D). These findings strongly suggest that iNKT cells control the cellularity and functions of ILC3s.

Our previous study demonstrated that CD1d-dependent iNKT cells play a significant role in controlling DSS-induced colitis in Yeti mice [4]. Moreover, it has been reported that iNKT cells can interact with CD1d-expressing ILC3s and consequently activate ILC3s to produce IL22 [15]. Thus, we tested whether iNKT cells can affect the activation of ILC3s in Yeti mice during DSS-induced colitis. We found that iNKT cell deficiency in Yeti/CD1d KO mice leads to a significant decrease in splenic and MLN ILC3 cell numbers (Figure 2E–G). Furthermore, we found that CD1d deficiency in Yeti/CD1d KO mice causes a dramatic reduction in ILC3s that produce IL22 (Figure 2H,I). These results were supported by additional experiments where spontaneous colitis was induced in Yeti mice housed under conventional, rather than barrier, conditions (Figure S3). Taken together, these results indicate that iNKT cells play critical roles in up-regulating the frequency and regulatory activity of ILC3s in Yeti mice.

### 2.3. Suppressive Effects of iNKT Cells on IFNγ-Mediated Intestinal Inflammation Are Closely Associated with Increased IL22 Production by ILC3s

**Figure 3:**
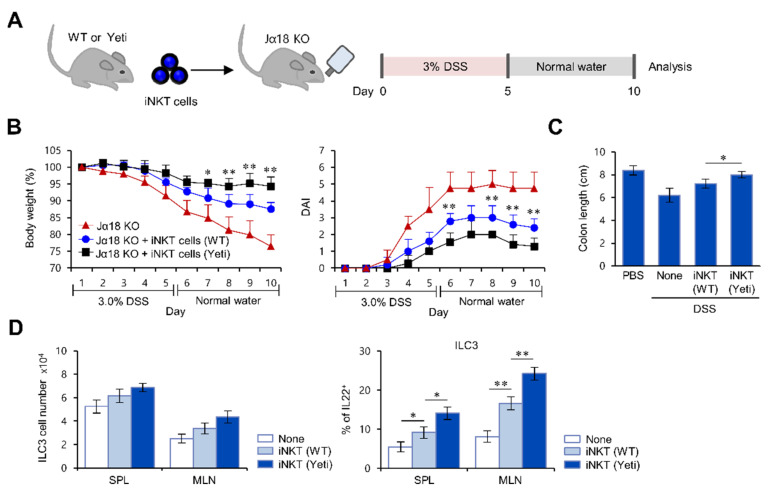
The suppressive effects of iNKT cells on IFNγ-mediated intestinal inflammation are closely associated with increased IL22 production by ILC3s. (**A**) Jα18 KO mice adoptively transferred with either WT or Yeti MLN iNKT cells (2 × 10^5^) were treated with 3% DSS as described in Materials and Methods. (**B**) Daily body weight changes, DAI score, and (**C**) colon length of these mice were evaluated. (**D**) The absolute numbers and IL22 production by ILC3s (MHC II^+^RORγt^+^Lin^−^) in the spleen and MLN from these mice were determined by flow cytometry on day 10. The mean values ± SD (*n* = 3; per group in the experiment; Student’s *t*-test; * *p* < 0.05, ** *p* < 0.01) are shown.

Next, to test whether suppression of colitis by iNKT cells correlates with IL22-producing ILC3s in Yeti mice, we performed adoptive transfer experiments to investigate the cooperative relationship between iNKT cells and ILC3s in colitis suppression. We adoptively transferred MLN iNKT cells (enriched using NK1.1^+^ iNKT cell isolation kit) from either WT or Yeti donor mice into iNKT cell-deficient Jα18 KO recipient mice (Figure 3A). Upon 3% DSS treatment, Jα18 KO mice that received Yeti iNKT cells displayed significantly reduced colitis severity compared to Jα18 KO mice that received WT iNKT cells (Figure 3B,C). From this set of experiments, we conclude that CD1d-dependent iNKT cells from Yeti mice play a significant role in controlling DSS-induced colitis.

Next, we considered that iNKT cells might affect ILC3 functions during colitis. To address this issue, we compared the effects of MLN iNKT cells purified from either WT or Yeti mice on the activation of ILC3s during DSS-induced colitis. Transfer of Yeti iNKT cells resulted in a modest (or insignificant) increase in total ILC3 cell numbers but a significant increase of IL22-producing ILC3s in the spleen and MLN of Jα18 KO recipient mice after DSS treatment. This finding strongly suggests that iNKT cells are essential players regulating ILC3 activation in Yeti mice (Figure 3D). Taken together, our results demonstrate for the first time that the crosstalk between iNKT cells and ILC3s plays a critical role in suppressing DSS-induced colitis in Yeti mice.

### 2.4. The IL9-Producing Subset of iNKT Cells Up-Regulates IL22-Producing ILC3s in IFNγ-Mediated Intestinal Inflammation

**Figure 4:**
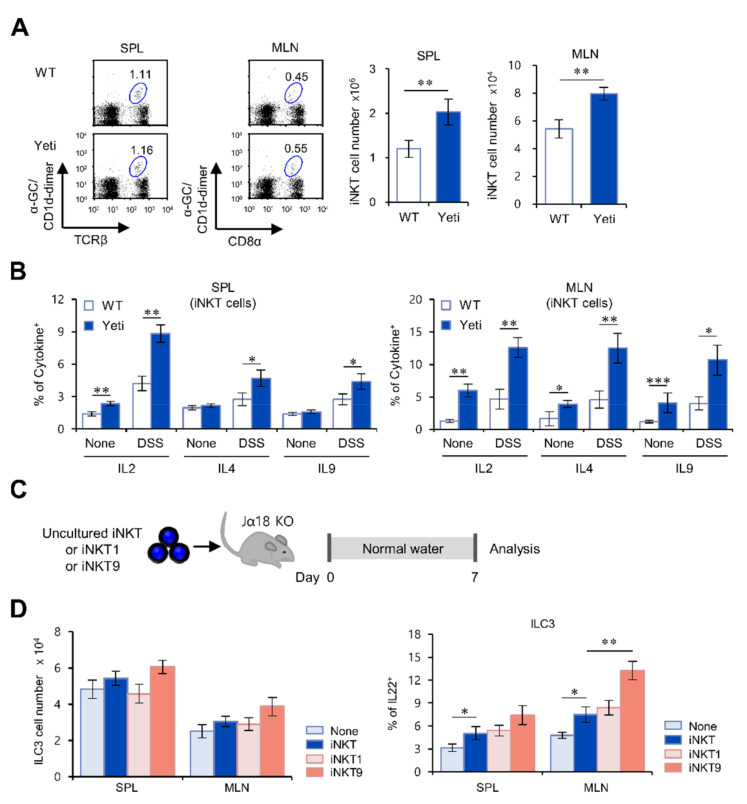
The IL9-producing iNKT cell subset up-regulates IL22-producing ILC3s in IFNγ-mediated intestinal inflammation. (**A,B**) WT and Yeti mice were treated with 1.5% DSS as described in Materials and Methods. The spleen and MLN were collected at ten days after DSS treatment. (**A**) The absolute numbers of iNKT cells in the spleen and MLN from WT and Yeti mice were measured by flow cytometry using α-GalCer-loaded CD1d dimers. Left, representative FACS plots; right, summary. (**B**) The spleen and MLN were obtained from DSS-treated or untreated WT and Yeti mice. Subsequently, the intracellular expression of IL2, IL4, and IL9 by splenic and MLN iNKT cells was determined by flow cytometry. (**C,D**) Sorted MLN iNKT cells from WT mice cultured under iNKT1 (rIL12, rIL2, and anti-IL4) or iNKT9 (rIL4, rTGFβ, rIL2, and anti-IFNγ) conditions. Either iNKT, iNKT1, or iNKT9 (2 × 10^5^) cells were IV transferred to Jα18 KO mice. The absolute numbers and IL22 production by ILC3s (MHC II^+^RORγt^+^Lin^−^) in the spleen and MLN from these mice were measured by flow cytometry at 7 days after injection. The mean values ± SD (*n* = 3 in **C** and **D**; *n* = 4 in A and B; per group in the experiment; Student’s *t*-test; * *p* < 0.05, ** *p* < 0.01, *** *p* < 0.001) are shown.

Since it has been demonstrated that IL9-producing iNKT cells provide protective effects against DSS colitis in an IL4-dependent manner [11], we decided to examine whether this subset of iNKT cells cooperates with IL22-producing ILC3s in regulating colitis in Yeti mice. In both DSS-treated and untreated mice, IL2-, IL4-, and IL9-producing iNKT cells in the spleen and MLN were significantly increased in Yeti mice compared with WT mice (Figure 4A,B). These results suggest that iNKT cells producing anti-inflammatory cytokines (especially IL9) are associated with ILC3 activation in Yeti mice during DSS-induced colitis. Thus, to determine whether IL9-producing iNKT cells promote ILC3 activation in our experimental setting, we prepared in vitro differentiated iNKT cells as follows: IL9-producing iNKT9 cells induced by recombinant IL4 (rIL4), rTGFβ, rIL2, and anti-IFNγ; IFNγ-producing iNKT1 cells induced by rIL12, rIL2, and anti-IL4; and non-polarized total iNKT cells as control. We adoptively transferred these in vitro differentiated/expanded iNKT cells into Jα18 KO mice and found that iNKT9 cells significantly enhanced IL22 production by ILC3s in the MLN compared with iNKT1 or non-polarized iNKT cells (Figure 4C,D). Collectively, these data reveal a functional link between IL9-producing iNKT cells and IL22-producing ILC3s in preventing intestinal inflammation in Yeti mice.

## 3. Discussion

A previous study demonstrated that MLN ILC3s participate in suppressing effector CD4^+^ T cells, but not Treg cells, via presenting microbiota-derived antigens on their MHC II molecules [12]. Moreover, ILC3s can present lipid antigens to iNKT cells via cell surface CD1d [15]. Here, we demonstrated that deficiency of iNKT cells in Yeti/CD1d KO mice causes a decrease in IL22-producing ILC3s. Thus, our findings provide strong evidence that increased IL22 production by ILC3s in the presence of iNKT cells suppresses antigen-specific IFNγ-producing CD4^+^ cells in the MLN of Yeti mice.

We found that ILC3s in the MLN are regulated by iNKT cells both during steady-state conditions and following DSS-induced colitis. MLNs are a crucial immune inductive site of the mucosal immune system, where non-invasive commensal bacteria are delivered by CX_3_CR1^hi^ phagocytic cells, resulting in T and B cell immune responses even in the absence of Myd88 signaling [20]. ILC3s are abundant in the MLN, possibly to control excessive inflammatory responses. During helminth infection, protective Th2 responses are also primarily restricted to the MLN [21]. Moreover, the distinct subsets of iNKT cells show a unique tissue distribution, reflecting their specialized regulatory functions in the particular organ. For instance, IV injection of α-GalCer preferentially activates iNKT1 cells in the spleen and liver, whereas oral administration of α-GalCer selectively activates iNKT2 populations (which likely contain IL9-producing iNKT cells) in the MLN [22]. Based on previous studies, including our own results, immune responses in the MLN should be tightly regulated to avoid uncontrolled inflammation. ILC3s alone might be insufficient to accomplish this goal and require assistance from iNKT cells producing anti-inflammatory cytokines such as IL4 and IL9. Thus, both ILC3s and iNKT cells contribute to maintaining homeostatic intestinal immune responses.

It has been reported that phosphorylated-STAT3 (p-STAT3) is required for RORγt expression in CD4^+^ T cells, and that p-STAT3 directly binds to the IL22 locus to regulate IL22 production by ILC3s [23]. Our data showed that iNKT cell-derived IL9 might be a critical cytokine to increase IL22 production by ILC3s. Based on a previous report that IL9 treatment induces STAT3 phosphorylation in colonic epithelial cells [24], further studies are warranted to assess the effect of iNKT cell-derived IL9 on STAT3 phosphorylation of ILC3s during IFNγ-mediated intestinal inflammation. Moreover, IL22 has been reported to relieve sepsis-induced liver injury via activating the JAK/STAT3 signaling pathway [25], suggesting that IL22-producing ILC3s exert regulatory functions on damaged tissues other than the intestine. Interestingly, ILC3s may promote carcinogenesis by inhibiting allergic immune responses in the skin [26], which illustrates the diverse functions of these cells. Thus, a better understanding of the crosstalk between IL9-producing iNKT cells and IL22-producing ILC3s will be fundamental for controlling inflammatory diseases in various organs, including the skin, liver, and intestine.

It has previously been demonstrated that iNKT cells activate ILC1s to secrete IFNγ and stimulate ILC2s to produce IL5 during influenza infection in the lung [27,28]. However, it is currently unknown whether the interaction of iNKT cells with either ILC1s or ILC2s requires direct engagement by iNKT TCR-CD1d ligation to ultimately contribute to modulating colitis. Thus, it will be informative to further investigate the contribution of iNKT cells to the activation of ILC1s and ILC2s in the gut during intestinal inflammation in Yeti mice.

Colitis increases the expression of the human nonclassical MHC class I molecules MICA and MICB on intestinal epithelial cells, resulting in the migration of CD8^+^ T cells into the inflamed intestine [29,30]. Furthermore, our previous report provided evidence that increased numbers of NK1.1^+^CD8^+^ T cells expressing NKG2D might cause damage to NKG2D ligand-expressing intestinal epithelial cells in Yeti mice [4]. Furthermore, IL22 treatment suppresses high fat diet-induced colonic epithelial cell stress and restores intestinal barrier function [31]. In future studies, it will be worthwhile to examine whether ILC3-derived IL22 can regulate NK1.1^+^CD8^+^ T cells, which are pathogenic effector cells in Yeti mice.

In conclusion, our results provide evidence that iNKT cells contribute to protection against IFNγ-mediated colitis by cooperating with ILC3s. Moreover, IL9-producing iNKT cells might be closely linked with the up-regulation of IL22-producing ILC3s. These findings identify the MLN iNKT–ILC3 regulatory axis as a novel target for immune therapy of IBD that may be achieved by oral administration of glycolipids.

## 4. Materials and Methods

### 4.1. Mice and Reagents

WT B6 mice were purchased from Jung Ang Lab Animal Inc. (Seoul, Korea). IFNγ/YFP (Yeti) cytokine reporter B6 mice were kindly provided by Dr. R. Locksley (University of California at San Francisco, CA, USA). CD1d KO B6 mice were provided by Dr. A. Bendelac (University of Chicago, IL, USA). Jα18 KO B6 mice were provided by Dr. M. Taniguchi (RIKEN, Yokohama, Japan). Yeti mice were further crossed with CD1d KO mice to obtain Yeti/CD1d KO mice. In this study, all mice were on a B6 genetic background, maintained at Sejong University, and used for experiments at 6–12 weeks of age. They were maintained on a 12 h light/12 h dark cycle in a temperature-controlled facility with free access to food and water. Most of the animals were maintained in autoclaved cages, fed γ-irradiated sterile chow, and provided with autoclaved tap water. For the studies evaluating spontaneous colitis development, mice were housed in conventional cages, fed non-sterile chow, and provided with normal tap water. Age- and sex-matched mice were used for all experiments. The animal experiments were approved by the Institutional Animal Care and Use Committee at Sejong University (SJ-20160704, 7-22-2016). α-GalCer was purchased from Enzo Life Science (Farmingdale, NY, USA).

### 4.2. Induction of Colonic Inflammation

Mice were provided with 1.5 (*w*/*v*) DSS in the drinking water for 5 days. Subsequently, groups of mice were given regular water for 5 days until sacrifice for experiments. To evaluate the clinical symptoms of DSS-induced colitis, the mice were monitored for a change in the percentage of body weight (0, none; 1, 1–10%; 2, 11–20%; 3, >20%), stool consistency (0, normal; 1, loose stool; 2, diarrhea), and bleeding (0, normal; 1, hemoccult positive; 2, gross bleeding) on a daily basis during colitis induction for 10 days. The body weight was expressed as a percentage of weight change for each individual mouse and was calculated relative to the starting body weight on day 0. These data were used to calculate a disease activity index (DAI).

### 4.3. Cell Culture and Cell Enrichment by Magnetically Activated Cell Sorting(MACS)

A single-cell suspension of splenocytes was prepared and resuspended in RPMI complete medium, consisting of RPMI 1640 (Gibco BRL, Gaithersburg, MD, USA) medium supplemented with 10% FBS, 10 mM HEPES, 2 mM L-glutamine, 100 units/mL penicillin–streptomycin, and 5 mM 2-mercaptoethanol. iNKT cells were enriched using an NK1.1^+^ iNKT cell isolation kit (Miltenyi Biotech, Bergisch Gladbach, Germany) following the manufacturer’s instructions [18]. The NKT cell population was >89% pure among all MACS-purified populations.

### 4.4. CD1d/α-GalCer Dimer Staining for iNKT Cells

To stain iNKT cells specifically, mCD1d/Ig fusion proteins (CD1d dimer; mouse CD1d dimerX, BD Biosciences, San Jose, CA, USA) were incubated overnight at 37 °C with a 40-fold molar excess of α-GalCer (in PBS containing 0.5% Tween 20). The staining cocktail was prepared by mixing α-GalCer-loaded mCD1d/Ig proteins with FITC- or APC-conjugated anti-mouse IgG1 Ab (clone A85-1, BD PharMingen, San Diego, CA, USA) at a 1:2 ratio of dimer to anti-mouse IgG1 Ab. Subsequently, the mixture was incubated for 2 h at room temperature.

### 4.5. In Vitro iNKT Cell Differentiation

MACS-sorted MLN iNKT cells from WT mice were cultured with plate-bound anti-CD3ε (10 μg/mL) + anti-CD28 (1 μg/mL) monoclonal antibodies (mAbs) in the presence of either iNKT9-polarizing conditions (rIL4 (10 ng/mL), rTGFβ (10 ng/mL), rIL2 (10 ng/mL), and anti-IFNγ (5 μg/mL)) or iNKT1-polarizing conditions (rIL12 (10 ng/mL), rIL2 (10 ng/mL), and anti-IL4 (5 μg/mL)) for 3 days.

### 4.6. Flow Cytometry

The following mAbs were obtained from BD Biosciences (San Jose, CA, USA): phycoerythrin (PE)- or allophycocyanin (APC)-conjugated anti-NK1.1 (clone PK-136), PE-Cy7- or APC-conjugated anti-CD11c (clone HL3), PE-conjugated anti-MHC II (clone M5/114.15.2), PE-Cy7-conjugated anti-TCRβ (clone H57-597), PE-Cy7-conjugated anti-CD8α (clone 53-6.7), PE-Cy7- or APC-conjugated anti-CD3ε (clone 145-2C11), APC-conjugated anti-CD19 (clone ID3), PE-Cy7- or APC-conjugated anti-CD11b (clone M1/70), APC-conjugated anti-B220 (clone RA3-6B2), PE-conjugated anti-IL12p40 (clone C15.6), PE-conjugated anti-TNFα (clone XP6-XT22), PE-conjugated anti-IL6 (clone MP5-20F3), PE-conjugated anti-IL2 (clone JES6-5H4), PE- or PerCP-CyTM5.5-conjugated anti-RORγt (clone Q31-378), PE-conjugated anti-IL4 (clone BVD6-24G2), PE-conjugated anti-IL9 (clone D9302C12), and PE-conjugated anti-IgG1 (κ isotype control). The following mAbs from eBioscience (ThermoFisher Scientific, Waltham, MA, USA) were used: APC-conjugated anti-FcεRIα (clone MAR-1), APC-conjugated anti-Ly-6G (Gr-1) (clone 1A8-Ly6G), APC-conjugated anti-F4/80 (clone BM8), PE-conjugated anti-IL22 (clone 1H8PWSR). Flow cytometric data were acquired with a FACSCalibur system (Becton Dickinson, San Jose, CA, USA) and analyzed with FlowJo software (version 8.7; Tree Star, Ashland, OR, USA). For surface antibody staining, cells were harvested and washed twice with cold 0.5% BSA-containing PBS (FACS buffer). For blocking non-specific binding to Fc receptors, the cells were incubated with anti-CD16/CD32 mAbs on ice for 10 min and subsequently stained with fluorescently labeled mAbs. Flow cytometric data were acquired using a FACSCalibur flow cytometer (Becton Dickson, San Jose, CA, USA) and analyzed using FlowJo software (version 8.7; Tree Star, Ashland, OR, USA).

### 4.7. Intracellular Cytokine Staining

For intracellular staining, splenocytes were incubated with brefeldin A, an intracellular protein transport inhibitor (10 μg/mL), in RPMI medium for 2 h at 37 °C. The cells were stained for cell surface markers, fixed with 1% paraformaldehyde, washed once with cold FACS buffer, and permeabilized with 0.5% saponin. The permeabilized cells were then stained for an additional 30 min at room temperature with the indicated mAbs (PE-conjugated anti-IL2, anti-IL4, anti-IL9, and anti-IL22; PE-conjugated isotype control rat IgG mAbs). Fixation and permeabilization were performed using a Foxp3 staining kit (eBioscience) with the indicated mAbs (PE-conjugated anti-RORγt; PE-conjugated isotype control rat IgG mAbs). More than 5000 cells per sample were acquired using a FACSCalibur, and the data were analyzed using the FlowJo software package (version 8.7; Tree Star, Ashland, OR, USA).

### 4.8. Isolation of Colon MLN Leukocytes

MLN were aseptically removed, and single-cell suspensions were generated by homogenization and passing through a 70 μm nylon cell strainer. The large intestines were removed and flushed with 20 mL of cold CMF solution (Ca^2+^–Mg^2+^-free PBS containing 10 mM HEPES, 25 mM sodium bicarbonate, and 2% FBS).

### 4.9. Histology

Distal colonic sections were fixed in 4% paraformaldehyde, embedded in paraffin, and cut into 6 μm sections using a microtome (RM 2235, Leica, Germany). The sections were then stained with H&E for analysis of histological changes.

### 4.10. Statistical Analysis

Statistical significance was determined using Excel (Microsoft, USA). Student’s *t*-test was performed for comparing two groups. In the Student’s *t*-test, * *p* < 0.05, ** *p* < 0.01, and *** *p* < 0.001 were considered to be significant. Two-way ANOVA analysis was carried out using VassarStats (http://vassarstats.net/anova2u.html). In the two-way ANOVA, # *p* < 0.05, ## *p* < 0.01, and ### *p* < 0.001 were considered to be significant.

## Data Availability

The data will be available from the corresponding author on reasonable request.

## Supplementary Materials

The following are available online at https://www.mdpi.com/1422-0067/22/3/1250/s1, Figure S1: The CD8^+^ T cell population is largely responsible for the pathogenesis of colitis in Yeti mice, Figure S2: Yeti mice with dysregulated expression of IFNγ show balanced cytokine profiles when subjected to low-dose DSS-induced colitis, Figure S3: A decrease of ILC3s correlates with spontaneous intestinal inflammation in Yeti/CD1d KO mice.
