# Supplementary material for: CD1d-Dependent iNKT Cells Control DSS-Induced Colitis in a Mouse Model of IFNγ-Mediated Hyperinflammation by Increasing IL22-Secreting ILC3 Cells
Source: Int J Mol Sci. 2021 Jan 27;22(3):1250. doi: 10.3390/ijms22031250 (PMC7866066; doi:10.3390/ijms22031250)
Supplement: Supplementary file 1 [file ijms-22-01250-s001.pdf]

Supporting documents to:

CD1d-dependent iNKT Cells Control DSS-Induced Colitis in a Mouse Model of IFNγ-mediated Hyperinflammation by Increasing IL22-Secreting ILC3 Cells

This document includes:

-Supplementary figures 1-3

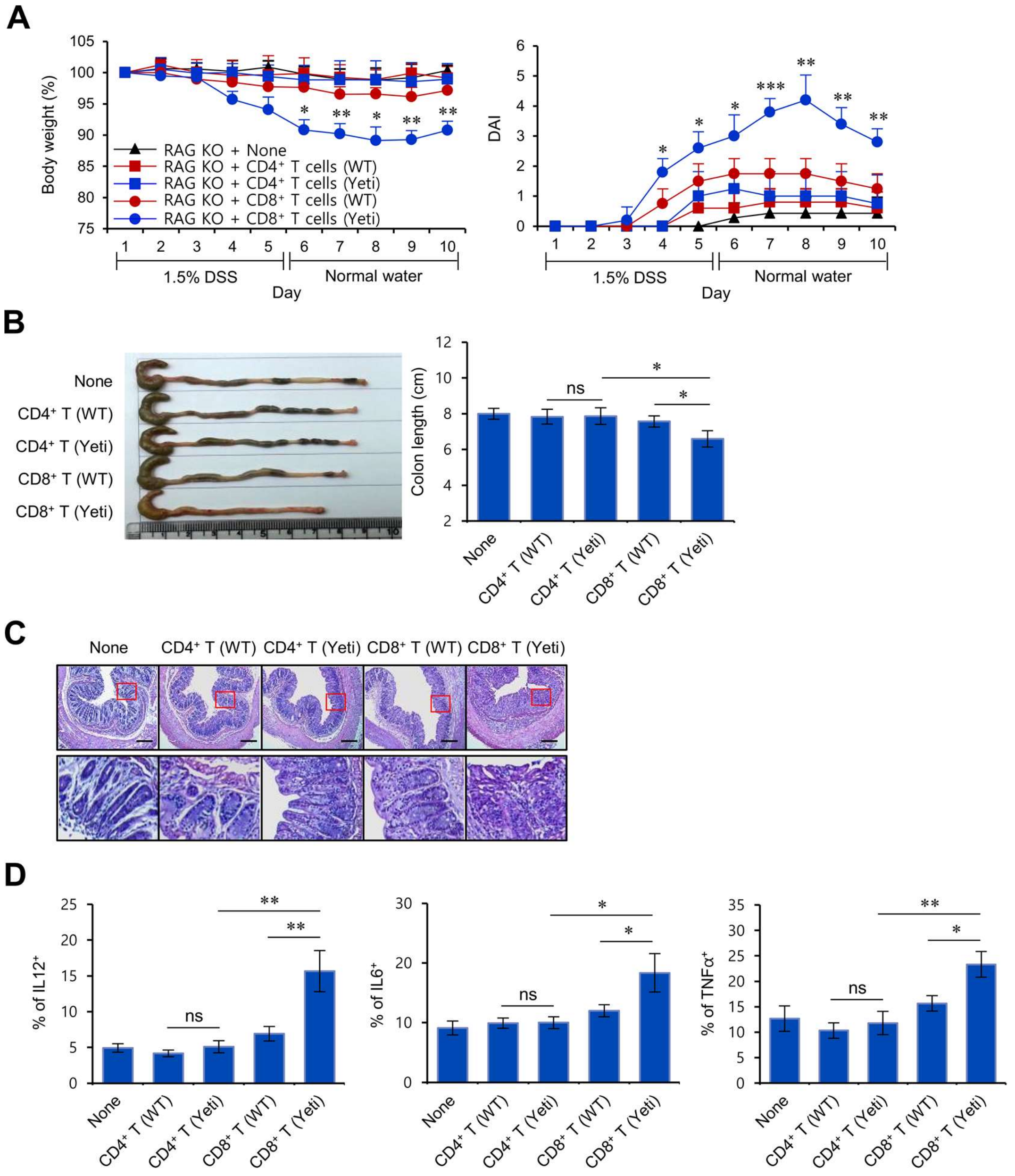

Figure S1. The CD8$^+$ T cell population is largely responsible for the pathogenesis of colitis in Yeti mice.

(A-D) MLN CD4$^+$ T cells (2×10^6) or MLN CD8$^+$ T cells (2×10^6) purified from WT or Yeti mice were i.v. transferred to RAG KO mice. Daily body weight changes, disease activity index (DAI) score (A), and colon length (B) of each group of animals were evaluated ten days after 1.5% DSS treatment. (C) Distal colon from each group was sectioned and stained with H&E on day 10. (D) The intracellular expression of IL12, IL6, and TNFα by MLN CD11c$^+$ DCs was analyzed by flow cytometry on day 10. The mean values ± SD (*n* = 5 per group in the experiment; Student's t-test; *P<0.05, **P<0.01) are shown.

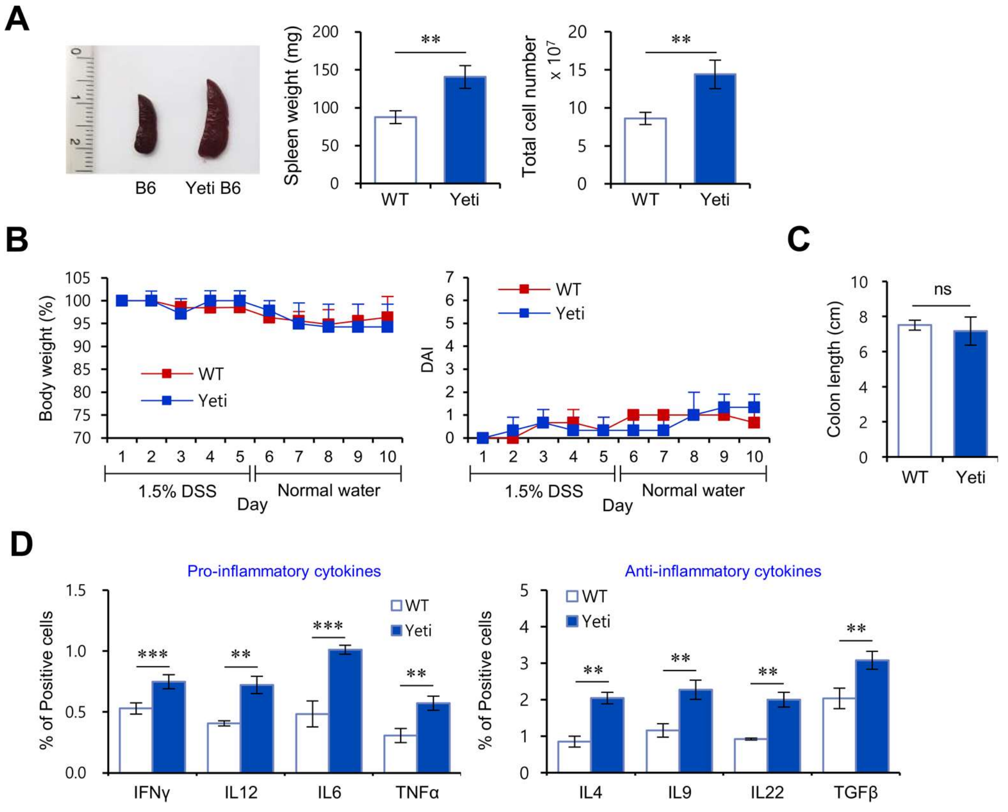

Figure S2. Yeti mice with dysregulated expression of IFNγ show balanced cytokine profiles when subjected to low dose DSS-induced colitis.

(A) *Left*, Representative picture of the spleens from 8-week-old WT and heterozygous Yeti mice. *Middle and Right*, Spleen weight and splenocyte numbers in Yeti mice compared with WT mice. Daily body weight changes, DAI score (B), and colon length (C) of WT and Yeti mice were evaluated after colitis induction by 1.5% DSS treatment. Data are representative of three independent experiments with similar results. (D) Intracellular production of inflammatory cytokines (IFNγ, IL12, IL6, and TNFα) and anti-inflammatory cytokines (IL4, IL9, IL22, and TGFβ) by MLN cells from WT and Yeti mice was assessed by flow cytometry at ten days after 1.5% DSS treatment. The mean values ± SD (n = 4; per group in the experiment; Student's t-test; *P<0.05, **P<0.01, ***P<0.001) are shown.

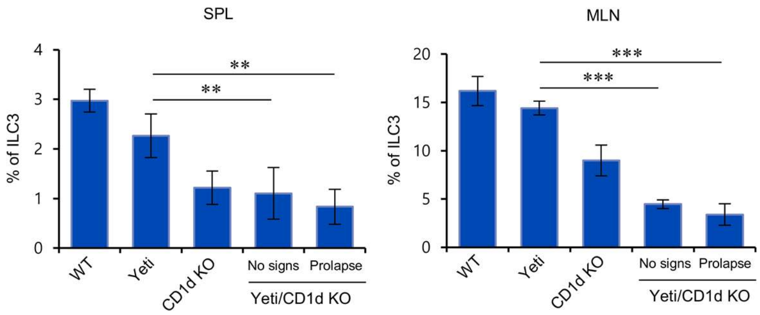

Figure S3. A decrease of ILC3s correlates with spontaneous intestinal inflammation in Yeti/CD1d KO mice.

WT, Yeti, CD1d KO, and Yeti/CD1d KO mice were housed for 12 weeks under conventional conditions, starting from 6 weeks of age. The spleen and MLNs were collected at 12 weeks after conventional housing conditions. (B) The absolute numbers of ILC3s (MHC $II^{+}ROR\gamma t^{+}Lin^{-}$) in the spleen and MLN from WT and Yeti mice were assessed by flow cytometry. The mean values ± SD (*n* = 5; per group in the experiment; Student's t-test; **P<0.01, ***P<0.001) are shown.
